# Accurately Inferring Compliance to Five Major Food Guidelines Through Simplified Surveys: Applying Data Mining to the UK National Diet and Nutrition Survey

**DOI:** 10.2196/publichealth.9536

**Published:** 2018-05-30

**Authors:** Nicholas Rosso, Philippe Giabbanelli

**Affiliations:** ^1^ Data Analytics for Complex Human Behaviors Laboratory Computer Science Department Northern Illinois University DeKalb, IL United States; ^2^ Data Analytics for Complex Human Behaviors Laboratory Department of Computer Science Furman University Greenville, SC United States

**Keywords:** diet, food, and nutrition, public health informatics, supervised machine learning

## Abstract

**Background:**

National surveys in public health nutrition commonly record the weight of every food consumed by an individual. However, if the goal is to identify whether individuals are in compliance with the 5 main national nutritional guidelines (sodium, saturated fats, sugars, fruit and vegetables, and fats), much less information may be needed. A previous study showed that tracking only 2.89% of all foods (113/3911) was sufficient to accurately identify compliance. Further reducing the data needs could lower participation burden, thus decreasing the costs for monitoring national compliance with key guidelines.

**Objective:**

This study aimed to assess whether national public health nutrition surveys can be further simplified by only recording whether a food was consumed, rather than having to weigh it.

**Methods:**

Our dataset came from a generalized sample of inhabitants in the United Kingdom, more specifically from the National Diet and Nutrition Survey 2008-2012. After simplifying food consumptions to a binary value (1 if an individual consumed a food and 0 otherwise), we built and optimized decision trees to find whether the foods could accurately predict compliance with the major 5 nutritional guidelines.

**Results:**

When using decision trees of a similar size to previous studies (ie, involving as many foods), we were able to correctly infer compliance for the 5 guidelines with an average accuracy of 80.1%. This is an average increase of 2.5 percentage points over a previous study, showing that further simplifying the surveys can actually yield more robust estimates. When we allowed the new decision trees to use slightly more foods than in previous studies, we were able to optimize the performance with an average increase of 3.1 percentage points.

**Conclusions:**

Although one may expect a further simplification of surveys to decrease accuracy, our study found that public health dietary surveys can be simplified (from accurately weighing items to simply checking whether they were consumed) while improving accuracy. One possibility is that the simplification reduced noise and made it easier for patterns to emerge. Using simplified surveys will allow to monitor public health nutrition in a more cost-effective manner and possibly decrease the number of errors as participation burden is reduced.

## Introduction

Insufficient compliance with dietary guidelines can lead to several health problems, whereas following guidelines can have protective effects. Systematic reviews have linked excess salt consumption with increased blood pressure, which raises the risk for cardiovascular diseases [1,2]. Furthermore, other meta-reviews have found that a higher consumption of fruit and vegetables “was significantly associated with a lower risk of all-cause mortality” [3]. It is, thus, essential to monitor compliance with such guidelines to understand and improve a population’s health. To assess whether guidelines are followed, data on nutritional intake must be compiled. A comprehensive assessment of nutritional intake can be burdening (as individuals need to record the exact amount and type of foods consumed), which may in part cause the inaccuracies found when individuals provide such reports [4].

Data mining is a computational technique (often equated with machine learning), which offers significant potential to alleviate that burden by finding key patterns in data. Among the different tasks performed in data mining, our focus is on *classification*, which consists of automatically relating a set of feature variables (eg, age, gender, food consumed) to an outcome (eg, being in compliance with guidelines on salt). Classification has been increasingly used in recent years for research on several weight-related outcomes, such as obesity [5-7], nutrition [8], and physical activity [9].Classification has demonstrated its potential to complement statistical regressions, particularly for nonlinear phenomena (as is often the case with human behaviors [10] such as eating behaviors), and without requiring a priori assumptions on the relationship between patterns and outcomes [11]. In particular, classification has been applied on several occasions to find the key questions that surveys need to infer a target behavior. For instance, in the case of adolescent binge drinking, researchers showed that rules in a household were strongly linked with the outcome, whereas other dimensions (eg, communication) were not as salient [12]. Similarly, previous research in public health nutrition found that only 2.89% (113/3911) of the food items were required to infer compliance to the 5 major national guidelines [8].

**Figure 1:**
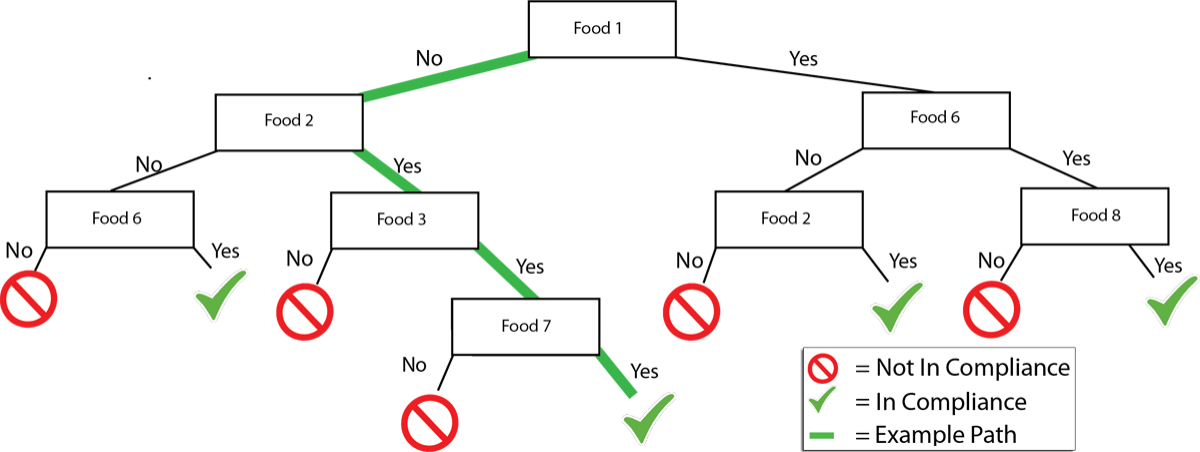
A decision tree starts at a root (top). For a given individual, we repeatedly compare the individual’s data with the questions in the tree. In this example, if the individual did not consume food 1, then the follow-up question is whether food 2 was consumed. Eventually, we reach a conclusion: whether the individual was in compliance with the guideline or not. Such trees are automatically built from the data.

There are many algorithms to choose from when performing classification. Decision trees in particular have proven to be a popular approach [6-9,11-13] for at least 2 reasons. First, they can then be used as a visual tool: instead of being a black-box model (such as a deep neural network or a support vector machine), they clearly articulate the rules that transform the description of a new participant’s case into an outcome (Figure 1). Second, these rules can also be used as flowcharts in public health, or clinical settings, to support decision-making activities (eg, triage) [14,15]. In line with these studies, this paper employs the classification technique of decision trees.

Our overarching goal is to further simplify public health nutrition surveys. Building on previous work showing that only 2.89% (113/3911) of the items were necessary [8] to infer compliance with major food guidelines, we will assess whether survey items can be reduced to binaries (was a food eaten or not?) rather than requiring an accurate weight. To identify *success* in adequately simplifying surveys, we will compute whether decision trees can still accurately infer compliance with guidelines using the simplified surveys. Specifically, we will simplify items in the National Diet and Nutrition Survey (NDNS) 2008-2012 to binary and assess whether decision trees built on the simplified dataset are about as accurate as decision trees built on the initial dataset.

The principal contributions of this study can be summarized as follows:

- We demonstrate that simplifying the information recorded in a specific dietary survey is not necessarily detrimental to identifying key public health outcomes.
- The application of our work to dietary public health suggests that nutritional surveys may be simplified when the aim is to predict compliance with major nutritional guidelines. This simplification may reduce participation burden, lower study costs, or increase the sample size at a same cost.
- The methodological part of our work illustrates the potential for data mining to contribute to public health not only by making predictions, but by identifying what part of the data is truly needed to form these predictions.

## Methods

### Data Used

Our dataset came from a generalized sample of inhabitants from the United Kingdom: the National Diet and Nutrition Survey (NDNS) 2008-2012. The NDNS data were obtained from the UK Data Archive [16]. The NDNS is a cross-sectional survey that records the nutrient intake as well as the nutritional status of the population within the United Kingdom [17]. To allow for comparison with previous studies [8], we used data from years 1-4 of this program, collected in 2008-2012. The NDNS collected data from a sample of 1000 respondents per year, consisting of adults and children aged 18 months and above. Households across the United Kingdom were selected to take part in the NDNS using a multistage probability design. During each wave, a random sample of primary sampling units was selected for inclusion. These are small geographical areas that allow for more efficient data collection by being geographically focused.

Within the dataset, food consumption at a daily level is recorded for participants over several days. To record portion sizes, common household measures (eg, one tablespoon, one cup) and weight in grams were used for the foods consumed throughout the study, including the consumption of liquids. Foods are described specifically and can be related to other foods in a subgroup or a group. For instance, the consumption of bananas would be entered as with 3 different levels of detail: as individual foods (eg, bananas raw flesh only), as subfood groups (eg, bananas), or as food groups (eg, fruit and vegetables).

The NDNS dataset only contains the foods consumed, their composition, and demographical information. It does not make any conclusion in regard to nutritional guidelines. The dataset was expanded in a previous study to include this information [8]. This was realized via the following process: (1) compute how much each individual consumed with respect to the 5 key dietary guidelines, then (2) compare this consumption with nutritional recommendations (which may be age-dependent), and (3) record the result as “Yes” when the participant was in compliance for a specific guideline or “No” when the participant was not in compliance. The detailed process is as follows.

The NDNS dataset has 4156 participants including 1189 children younger than 11 years. First, for each of the 4156 participants, compute the mean daily intake of fruit and vegetables and sodium, as well as the main daily percentage of energy derived from fat, saturated fat, and free sugars. Then, compare each individual's numbers with the corresponding nutritional recommendations to determine whether the individual is in compliance with the recommendation. UK recommendations on *fruit and vegetables* apply only to those aged 11 years or older, thus 1189 participants were excluded for this specific comparison. To be recorded as “Yes,” those retained needed to consume at least five 80-g portions of fruit and vegetable daily, allowing for at most 1 portion of juice. Although UK recommendations on *sodium* are also dependent on the age category, they adjust the comparison rather than excluding participants. A participant would be labeled as “Yes” if the sodium intake does not exceed [18] 2400 mg/d for those aged 11 years and older, 2000 mg/d for those aged 7-10 years, 1200 mg/d for those aged 4-6 years, and 800 mg/d for those aged 1-3 years.

The World Health Organization (WHO) recommends limitations on how much energy can be derived from each of the following categories: at most 30% from fat, at most 10% from saturated fat, and at most 10% from free sugars (sixth table in [19]). We then computed how much energy a participant derived from each category. If the energy derived from fat, saturated fat, and free sugars were under the WHO threshold, then we set the corresponding guideline to “YES.”

For each participant, our final dataset includes selected data from the NDNS survey (age, gender, and consumption for all of the 3911 individual foods) and additional data computed through the process above (whether or not they were in compliance for each of the 5 nutritional guidelines).

### Methods Employed: Classification Using Decision Trees

A *classifier* is a model automatically built from a subset of the data (called *training set*) in which we know both the predictor variables (ie, age, gender, and foods eaten) and the class outcomes (ie, whether or not each of the 5 guidelines was met). The intention is to build “good” classifiers, that is, models that learn and generalize from the training set so that they can accurately predict the outcomes when presented with new cases [20]. Numerous methods build classifiers, such as support vector machines, decision trees, and rulesets [21]. As detailed in the Introduction, our study uses decision trees, which are a commonly used approach [6-9,11-13] that provides a usable visual tool (Figure 1) to support decision-making activities such as triage.

There are 2 types of classifications: binary and multi-class. In a binary situation, the outcome we seek to predict can only have 2 different values. Conversely, in a multi-classification problem, the outcome has 3 or more values. Our study focuses on a binary classification problem: for each of the 5 guidelines, we want to know whether or not the guideline is met.

The process to create a decision tree for binary classification has been detailed in numerous reference material such as Maimon and Rokach [22]; thus, we provide only a brief overview of this process. The dataset (detailed in the previous section) comes in as a spreadsheet, where rows correspond to individuals and columns represent their features (ie, their age, gender, diet, and whether or not each of the 5 guidelines was met). The goal is to train a decision tree so it automatically identifies the combination of predictor variables (age, gender, individual foods) to determine the class outcome (for each of the five guidelines). A small portion of the rows are used as the *training set* to guide the decision tree algorithms to produce specific trees. The algorithm will repeatedly subdivide the data, where the variable used to subdivide is represented as a node in the tree (Figure 1), and the subdivisions corresponding to different values are shown as branches leaving this node. For instance, Figure 1 shows that the first division is based on the hypothetical “food 1”: one subdivision is produced when the food was not consumed (left branch), and the other subdivision corresponds to consuming this food (right branch).

A portion of the data is not provided to the algorithm for building the tree and is instead held to evaluate the quality of the generated tree [20]. This portion is called the *testing set*. To avoid basing our evaluation from one specific portion of the data that may not be representative, a process known as *cross-fold validation* divides the dataset into multiple portions, building the tree on one (training) and evaluating it on the others (testing) before repeating the division until all parts have been used for training and testing. This common process to evaluate classification accuracy helps prevent overfitting, where performances on the training set are very good but its generalization on the training set performs poorly [20]. The evaluation consists of presenting the tree with individuals from the testing set and asking what the classes should be. Then, the tree predicts a class outcome, which we compare with the real outcome from the dataset. The extent to which these outcomes match is called the *accuracy*. When the outcomes are binary, the percentage of “Yes” instances correctly classified is known as *recall*, and the percentage of “No” instances correctly classified is known as *specificity*. Intuitively, accuracy is the performance of the model across class outcomes, whereas recall and specificity are performances for one outcome in particular.

Highlighting recall and sensitivity is useful when the costs of making mistakes may be different: in health studies, giving someone an intervention that they do not need may be a very different issue from initially suggesting not to give them the intervention that they need. In addition, datasets are frequently imbalanced, that is, there can be many more cases for one outcome than the other. In this case, a high accuracy may be misleading as the tree may do well for the most common case, while being very inaccurate for the less common case. By providing the recall and sensitivity, our study supports public health officials in evaluating our performance by giving more or less weight to specific outcomes. As in previous work, our overall accuracy assumes that the error costs are similar [8], that is, concluding that someone does not follow guidelines while they do is no worse than concluding that they follow guidelines while they do not. Assuming different error costs would need additional evidence, and it would also lead to different methods as relatively few approaches can mine data under differential error costs [23,24].

In general, class imbalance can be addressed by eliminating cases of the majority class (undersampling), creating new cases for the minority class (oversampling), or biasing the classification algorithm (eg, using nonuniform error costs on the classes) [12,24]. For this study, we use sampling techniques. Specifically, we used Synthetic Minority Over-Sampling Technique, or SMOTE for short. As concluded by Batista et al, “over-sampling methods in general, and SMOTE-based methods in particular” were very efficient to address class imbalances [5,25].Although a comprehensive discussion on class balancing is beyond the scope of this study, we note that finding good approaches for synthetic over-sampling remains a very active area of research, as even popular methods such as SMOTE have weaknesses. However, such weaknesses are particularly encountered when dealing with very high-dimensional datasets such as text [26], which is not the case here.

### Overall Process

**Figure 2:**
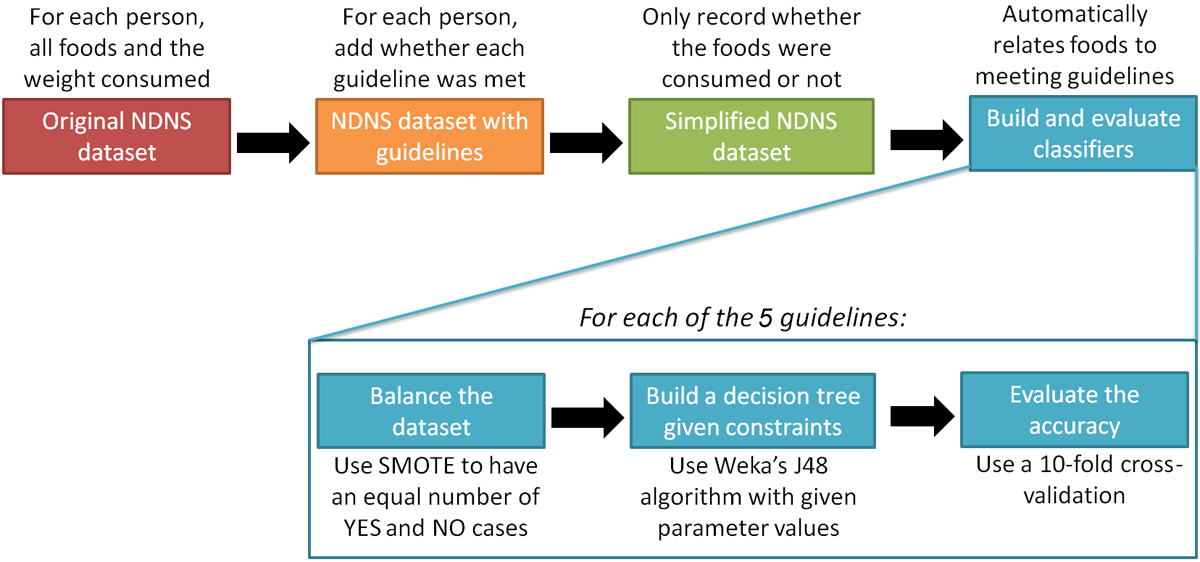
Flow diagram of our methodology, showing the acquisition, preprocessing, and mining of the data. NDNS: National Diet and Nutrition Survey; SMOTE: Synthetic Minority Over-Sampling Technique.

Our process is summarized in Figure 2. We start with the same dataset as used in our previous study: the NDNS 2008-2012 data expanded with compliance to each guideline [8].We departed from the previous study [8] by simplifying the dataset: we only recorded whether an item was consumed (1) or not (0). These data are given as input to the classification process, which was performed 5 times, for each of the guidelines. For a given guideline, we removed the compliance of the 4 other guidelines from the dataset. We do not want the algorithm to use compliance on fat to infer compliance on saturated fat: instead, compliance should be inferred from the foods, age, and gender only. As discussed in the previous subsection, balancing needs to be performed to avoid biasing the algorithm in favor of the most common outcome. We used SMOTE to ensure that both outcomes (meeting or not meeting a guideline) occur with the same prevalence. The balanced dataset was then fed into the Weka software version 3.7, maintained by the Machine Learning Group at the University of Waikato. We used the J48 decision tree algorithm, which implements the highly cited C4.5 algorithm by Ross Quinlan [27].

Like most classification algorithms, C4.5 (and its J48 implementations) take parameters that can impose further constraints on the resulting tree. We tested different parameter values to either (1) find the most accurate decision tree with a similar structure (ie, number of foods) to the trees generated in the previous study using the exact weights of foods, or (2) identify the most accurate tree without consideration for the number of foods involved [8]. These allow to perform two operations. First, we can *compare* with the previous study [8], in which the tree built for each guideline used a very small number of foods. Our objective was to constrain our new tree in using a similar number of foods, such that we can observe how accuracy changes when foods are simplified (in this study) instead of being recorded exactly (in the previous study). To lower the number of foods used by the algorithm, we increased the minimum number of cases required to further cut the data (ie, add a decision node to the tree). Second, we seek to *optimize*, by identifying how accurate we can be using our simplified foods, possibly at the expense of using more foods.

After each tree was built, we used 10-fold cross-validation [10,20,28]. This method for evaluation divides the dataset into 10 equal parts. Nine parts were used for the training set, and one for the testing set. After the process was repeated 10 times, the evaluation was conducted on all of the data, and the average results were reported. For full disclosure, all of our decision trees are available on the Open Science Framework platform (see [29]).

A sample of our approach to explore the trade-off between the number of foods and accuracy is illustrated in Table 1, showing a parameter sweep by increasing the minimum number of instances to (nonmonotonically) reduce the number of foods used. The rationale for this process is as follows. For the decision tree algorithm to create a new branch, it needs to find where to “cut” in the dataset. If there are not enough instances to cut, then a new branch will not be made. When this new branch *would* have been based on a factor not previously used in the tree, then preventing its creation limits the number of foods used. However, the branch may have involved an already existing factor. Raising the minimum number of instances thus limits opportunities for the algorithm to involve additional factors. Table 1 exemplifies how the number of factors tends to decrease as the minimum number of instances increases.

In the guiding example of Table 1, we predict adherence to the guideline on free sugars. The previous study used 28 foods for this class [8], thus we seek the highest accuracy that we can achieve with 28 foods or less. The best trade-off is found using a minimum of 95 instances, leading to 25 foods and an accuracy of 77.9%, which is higher than the 76.5% previously found. This trade-off would thus be reported in our results.

Table 1 exemplifies our methodology on choosing a decision tree comparable with the previous study [8], by changing the minimum number of instances. We observe that, as this number increases, the number of factors tends to decrease. The goal is to find the result with the highest accuracy while using no more foods than in the previous study.

**Table 1:** Sample outcome for the decision tree classifier on free sugars.

Study	Minimum number of instances	Accuracy (average)	Recall	Specificity	Number of factors
Previous	60	76.5	76.1	76.9	28
Current	60	78.2	73.6	82.9	31
Current	70	78.1	74.7	77.3	31
Current	80	78.3	74.7	78.3	30
Current	90	77.9	75.1	80.8	30
Current	95	77.9	75.1	80.7	25
Current	100	77.3	75.7	78.9	26
Current	115	77.2	75.5	78.8	22

## Results

**Figure 3:**
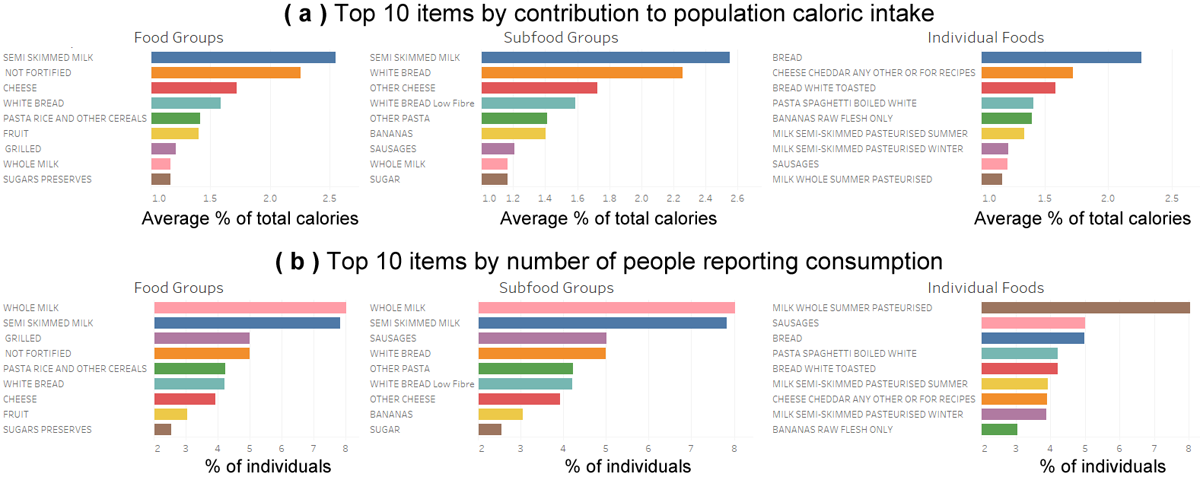
Main foods either by (a) contribution to caloric intake, or (b) prevalence among individuals.

Our dataset can broadly be understood as consisting of participants (the rows) and their food consumptions (the columns). Demographic characteristics of the participants (regardless of food consumptions) are summarized in Table 2 including gender, nationality, marital status, and economic status. Participants were on average 30.5 (SD 20.9) years old. Patterns of food consumption are shown in Figure 3. As will be shown in our results, it is not because a food is common that it should be included to identify whether participants meet a dietary guideline.

The methods introduced in the previous section select a food if it helps to separate individuals in compliance versus those who are not. For instance, if eating bananas is highly prevalent in the population, then knowing whether a person ate bananas may not be useful to predict dietary compliance. Conversely, if a food was clearly associated with a healthier diet for a handful of individuals, the frequency may be too low to warrant its inclusion at the population level.

Our new decision trees, built on simplified reporting of foods, were slightly more accurate than previous trees built using the exact weighted foods. This was found across all guidelines (Table 3). In 4 out of the 5 guidelines, the increase in accuracy was particularly noticeable to infer that someone did not meet a guideline. For instance, the previously reported accuracy of 78.4% [8] on finding noncompliance with fat had now increased to 88.5%. The increase in finding noncompliant cases was met in 2 guidelines (salt, free sugar) with a small decrease in accuracy for compliant cases, whereas it was similar in a third guideline (fat).

Across the 5 guidelines, our new decision trees had an accuracy of 80.1%. That is, in 4 out of 5 cases, by only knowing whether foods were consumed, and using at most a few dozen foods, we can successfully conclude whether nutritional guidelines are met. This accuracy is 2.6 percentage points higher than the average on previous decision trees (77.5%). That is, not asking individuals to weigh foods leads to being better able to tell if they meet guidelines.

**Figure 4:**
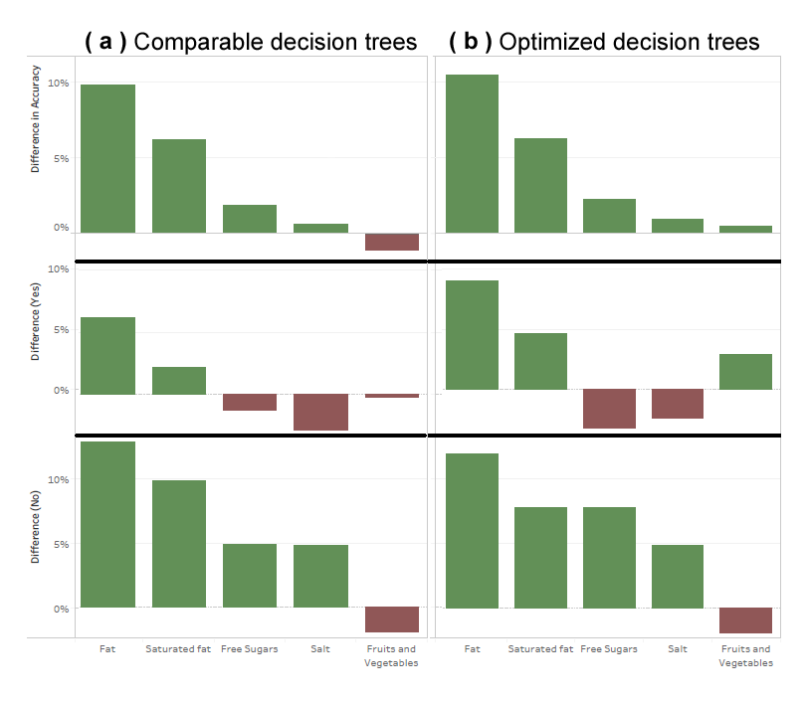
Accuracy, recall (“Yes”), and specificity (“No”) when (a) limiting the number of foods as in a previous study (Giabbanelli & Adams, 2016 [8]), or (b) using any number of foods to build the decision trees, giving us the optimized decision trees.

The optimized classifiers performed slightly better with an average accuracy of 80.6% on classified classes (Table 4). The optimized trees also had an average percentage increase of 3.1 points from the previous classifiers see (Figure 4). In all guidelines but one (salt), the increase in performance was obtained at the expense of using more foods. Although the number of foods used can increase by up to 50% (for saturated fat, fruits and vegetables), the absolute number of foods remains very small compared with the initial NDNS data and its 3911 foods.

To better contrast optimized decision trees versus those limited in the number of foods, Figure 4 shows where they led to either better (green) or lower (red) accuracy compared with the previous study [8]. Both methods generally underperformed on finding noncompliance to fruit and vegetables, and on finding compliance on salt and free sugars. They over-performed on fat and saturated fat. In summary, the consequences of simplifying dietary surveys are not uniform across guidelines, as some will see a small reduction in accuracy, whereas others may see a large improvement, resulting in the average accuracy (across all guidelines) being improved.

In Table 5, we list all individual foods used at least 5 times in predicting compliance with the guidelines, using either decision trees similar to the previous study [8], or the optimized trees. The expanded list of foods used one or more times is provided as supplementary material online [29]. Note that foods used to predict compliance with a guideline may not be part of what counts within this guideline. For instance, sausage rolls are neither fruit nor vegetables, yet they are used to predict fruit and vegetables consumption. We also observe that these foods are not necessarily the “common” ones shown in Figure 3.

**Table 2:** Key characteristics of the National Diet and Nutrition Survey (NDNS) household dataset. All participants in the study were within the United Kingdom. There were several study waves, with around 1000 respondents per year.

Characteristics		Categorical count, n (%)
**Gender**		
	Male	5034 (47.41)
	Female	5439 (52.57)
**Within compliance**		
	Free sugars	1472 (35.41)
	Salt	2524 (60.73)
	Fat	1045 (25.14)
	Saturated fat	795 (19.13)
	Fruits and vegetables	656 (15.78)
**Nationality**		
	English	5036 (48.08)
	Northern Irish	3442 (32.86)
	Scottish	684 (6.53)
	Welsh	398 (3.80)
	Irish	194 (1.85)
	Other	719 (6.88)
**Marital status**		
	Single (never married)	6240 (59.57)
	Married (living with partner)	1960 (18.71)
	Divorced	261 (2.49)
	Married (living separate)	3 (0.06)
	Widowed	139 (1.32)
	Other	1870 (17.85)
**Economic status**		
	Going to school full-time	2974 (28.39)
	Full or part time employment	4440 (42.39)
	Not working presently	3039 (29.02)

**Table 3:** Comparison of the best decision tree using the weight of foods (previous study, Giabbanelli and Adams, 2016 [8]) or simplified foods (this study), while keeping the number of foods similar.

Study	Guidelines	Number of instances	Accuracy (%)	Recall	Specificity	Number of factors
Previous	Free sugars	60	76.5	76.1	76.9	28
Current	Free sugars	95	77.9	75.1	80.7	25
Previous	Fat	70	72.4	66.3	78.4	33
Current	Fat	90	79.4	70.4	88.5	33
Previous	Fruits and vegetables	50	83.1	82.5	83.8	11
Current	Fruits and vegetables	90	82.2	82.3	82.2	10
Previous	Saturated fat	20	79.7	75.8	83.6	28
Current	Saturated fat	90	84.6	77.4	91.8	27
Previous	Salt	15	75.8	81.9	69.8	28
Current	Salt	55	76.3	79.5	73.2	26

**Table 4:** Comparison of the best decision tree using the weight of foods (previous study, Giabbanelli and Adams, 2016 [8]) or simplified foods (this study), without being limited by the number of foods.

Study	Guidelines	Number of instances	Accuracy (%)	Recall	Specificity	Number of factors
Previous	Free sugars	60	76.5	76.1	76.9	28
Current	Free sugars	60	78.2	73.6	82.9	31
Previous	Fat	70	72.4	66.3	78.4	33
Current	Fat	70	79.9	72.3	87.7	43
Previous	Fruits and vegetables	50	83.1	82.5	83.8	11
Current	Fruits and vegetables	50	83.5	84.9	82.2	16
Previous	Saturated fat	20	79.7	75.8	83.6	28
Current	Saturated fat	20	84.7	79.3	90.1	42
Previous	Salt	15	75.8	81.9	69.8	28
Current	Salt	50	76.6	79.9	73.2	25

**Table 5:** Individual foods used as predictors at least 5 times in the trees generated using our 2 processes (similar/optimized) and for the 5 guidelines: Fruit and Vegetables, Fat, Saturated Fat, Salt, and Free Sugars. The frequency is the number of times that a food is used as a decision node across all trees (eg, if used 3 times in 5 trees each, it would be 15).

Variables		Similar decision tree					Optimized decision tree					Total frequency
		FV^b^	Fat	SatFat^c^	Salt	Sug^d^	FV	Fat	SatFat	Salt	Sug	
**Individual food**												
	Sausages		✓	✓	✓			✓	✓	✓		20
	Bananas raw	✓	✓			✓	✓	✓	✓			19
	Sausage roll	✓	✓	✓			✓	✓	✓			16
	Cheese cheddar		✓	✓	✓			✓	✓	✓		14
	Milk chocolate		✓	✓				✓	✓			12
	Butter salted		✓		✓		✓		✓		✓	10
	Cheese spreads		✓						✓			8
	Ice cream		✓						✓		✓	8
	Fruit drink					✓			✓		✓	8
	Chicken pieces		✓					✓				8
	Sex		✓		✓					✓		7
	Potato crisps		✓		✓							6
	Apples	✓					✓					6
	Milk whole		✓	✓					✓			6
	Beans baked		✓		✓			✓		✓		6
	Onions	✓			✓							6
	Cola					✓					✓	6
	Apple juice unsweetened UHT^a^	✓				✓	✓				✓	6
	Olive oil	✓										6
	Orange juice unsweetened					✓					✓	6
	Orange juice unsweetened UHT										✓	6
	Bacon				✓							6
	Apple juice unsweetened						✓				✓	5
**Demographic**												
	Sex		✓		✓					✓		7

^a^UHT: Ultra-high-temperature processing.

^b^FV: fruits and vegetables.

^c^SatFat: saturated fat.

^d^Sug: free sugars.

## Discussion

### Principal Findings

Monitoring at the national level whether the population is in compliance with an array of nutritional guidelines currently requires an extensive data collection process, in which individuals report and weigh the exact foods that they consumed. Our previous study demonstrated that only 2.89% (113/3911) of the foods needed to be reported to predict with 77.5% accuracy (72%-83% across guidelines) whether individuals achieve key dietary recommendations regarding sodium, saturated fats, sugars, fruit/vegetables, and fats [8]. In this study, we investigated the consequences of further simplifying reporting by only asking participants whether they ate a specific food rather than having to weight it.

Although we may have expected a decreased accuracy as a consequence of removing information, our results paradoxically indicate that accuracy has improved to 80%. We observed that results were particularly improved when inferring compliance to the guidelines on fat and saturated fat, but a trade-off was operated on free sugars and salt where a decrease in recall was counter-balanced by a larger increase in specificity. Results were more nuanced on fruit and vegetables, where optimized decision trees were able to offset a loss of specificity with a higher gain in recall (thus resulting in higher accuracy), but nonoptimized decision trees resulted in a small loss of accuracy. Overall, these findings suggest that foods may not have to be weighted, but this may depend on (1) which food guidelines need to be monitored and (2) whether public health officials decide that recall is more important than sensitivity (or vice versa) instead of giving them equal weight.

The main applications of our results are twofold. First, we may simplify surveys not only by asking for few foods in adaptive questionnaires (as shown in [8]) but also by asking binary questions “Did you consume this food?” rather than requiring participants to provide an exact weight. This contribution will result in more time-effective assessments and may lower the cognitive effort required from participants, which in turn can decrease the error rate. Second, identifying a few questions yielding an accuracy of 80% is most applicable when a trade-off has to be found between accuracy and participation burden. For instance, a doctor may have many tools and physiological measures as part of the treatment process (eg, blood pressure, HbA1c), and including a few dietary questions with an accuracy of 80% may be more feasible than a more thorough survey. For population health, our work is particularly applicable in large studies where only a limited number of questions can be used to investigate a *subgroup within arms*. For instance, in the Netherlands, the nationwide Longitudinal Internet Studies for the Social sciences (LISS) panel sends questionnaires each month, dealing with many topics ranging from alcohol [30] to happiness. Nutrition would only be one part, and a reduced measurement approach would be necessary.

### Comparison With Other Dietary Methods

There are several alternatives to the analysis conducted here. First, an index-based analysis consists of a scoring system based on a priori knowledge that researchers have about (1) dietary guidance and (2) the scores to assign for sets of dietary components based on the guidance. This analysis can be used to assess adherence to guidelines [31-33] or summarize an individual's diet quality [2,31]. Within epidemiology, indices are used to identify the risk an individual will have to certain diseases based a combination of foods [31]. Although the reliance of indices on a priori knowledge makes them less sensitive to variations in the sample than our method, they may (depending on their design and structure) require more foods and accuracy in portion sizes. Considering the trade-off or “continuum” from few simple questions to favoring high accuracy, indices can lead to a higher accuracy than the method presented here but may not be as amenable to a “reduced” form as a short addendum to a large panel study such as LISS [30]. We also note that the transparency and simplicity of decision trees can support practitioners in interpreting the rules (eg, for triage) with little to no training, whereas dietary indices can produce summary scores where expertise is still important for interpretation.

Second, one could perform a cluster analysis. As summarized by Reedy et al, “clusters are driven by the sample from which they are derived, so their applicability as a standard for evaluating diets of different populations is limited because of the number of factors that determine food selection” [34]. Cluster analysis is an *unsupervised* data mining technique that identifies similarities between groups based on their patterns of food consumption: for instance, “fatty meats” may be an important similarity between men [34]. This is different from the classification approach taken here, which is a *supervised* data mining technique that seeks to predict an outcome.

Finally, Food Frequency Questionnaires (FFQs) can provide a cost-effective approach to monitoring the health of a large population. Molag et al [35], as well as Noethlings et al, suggested that portion sizes may not be necessary [36]: “We conclude that the omission of individual portion size information would probably result in a notable reduction of interindividual variance. However, to reduce the respondents' burden and to increase data completeness in self-administration in large epidemiologic studies, the assignment of a constant portion size seems to be adequate.” Our study confirms this finding while pointing out that accuracy may even increase; however, the effect depends on which guideline we monitor.

### Strengths and Limitations

Our study aimed to determine the effects of reducing the level of details employed by a national dietary survey. The NDNS survey used here has been the subject of many publications and provides a wealth of high-quality data. However, several limitations stem from using this survey. First, the NDNS survey relies on self-reported food intake. Individuals may consciously, or unconsciously, misreport their consumption within a 24-hour time frame [4,37,38]. Using the exact weigh of foods is thus sensitive to misreporting, which was a limitation of our previous study [8]. In contrast, this study is not sensitive to misreporting how much of a food was consumed: it only takes into account whether *any* consumption of this food occurred. Reporting errors affecting our study would thus be to entirely ignore a specific food that was consumed or to report a food that was not consumed.

Second, this survey was specific to the population of the United Kingdom, as can be seen in the specific foods used as predictors. This limitation of the data entails that our conclusion may not be generalized to populations that have important differences in eating behaviors. In this case, our approach can be replicated by collecting the complete dataset (in the first study wave) and then using data mining to investigate the consequences of simplifying it (for future study waves). Replicating results across target populations is necessary before concluding that monitoring compliance to nutritional guidelines may generally be simplified.

Our study used the data mining technique of decision trees to automatically relate individual food consumption to meeting specific guidelines. This is a well-researched technique, which has been applied to problems arising in health on multiple occasions. One specific advantage of decision trees lies in their ability to produce a model that can easily be interpreted and used with limited training. For instance, in triage, decision trees provide a “flowchart” that lay participants as well as field specialists can use intuitively. That is, an adaptive questionnaire can be formed by following the rules induced by a tree (Figure 1), which can be done using a computer program or by individuals. In contrast, many other techniques (eg, Support Vector Machines, Neural Networks) produce “black box” models, which are meant to be executed by machines rather than being read by humans. Future studies primarily concerned with accuracy (rather than transparency/readability of the model) may explore using such techniques. Contrasting the use of neural networks to decision trees over the same dataset would provide valuable insight on how accurate we can be without restrictions, which would help to better situate the results from this study.

### Conclusions

We sought to determine whether identifying individual dietary compliance can be further simplified while remaining as informative and accurate. We found that reporting very few foods and only whether they were consumed was sufficient to correctly identify compliance to 5 major nutritional guidelines. Being able to reduce the detail of a dataset for national monitoring can make it easier to increasing monitoring frequency or monitor more participants, thus increasing research participations without increasing study costs.

## Abbreviations

LISS: Longitudinal Internet Studies for the Social sciences
NDNS: National Diet and Nutrition Survey
SMOTE: Synthetic Minority Over-Sampling Technique
WHO: World Health Organization
